# Predictive factors for deep medial collateral ligament release in adjusted mechanical alignment total knee arthroplasty

**DOI:** 10.1186/s13018-024-05046-7

**Published:** 2024-09-28

**Authors:** Nimit Thongpulsawasdi, Chaiwat Achawakulthep, Tawan Intiyanaravut, Chirathit Anusitviwat, Varah Yuenyongviwat

**Affiliations:** 1 Golden jubilee medical center, Faculty of Medicine Siriraj hospital, Mahidol university, Nakhon Pathom, 73170 Thailand; 2 Department of Orthopedics, Faculty of Medicine, Prince of Songkla University, Hat Yai, Songkhla 90110 Thailand

**Keywords:** Robotic-assisted total knee arthroplasty, Adjusted mechanical alignment, Soft tissue balance, Osteoarthritis

## Abstract

**Background:**

Total knee arthroplasty (TKA) demands precision in achieving optimal alignment and soft tissue balance, especially in cases of medial compartment osteoarthritis where the need for medial soft tissue release is critical yet challenging to ascertain.

**Objective:**

This study aims to systematically investigate the relationship between preoperative data, initial knee conditions and the necessity for deep collateral ligament (MCL) release in adjusted mechanical alignment total knee arthroplasty.

**Methods:**

We conducted a retrospective study involving 61 TKA patients who underwent adjusted mechanical alignment robotic-assisted procedures. Soft tissue release was carried out when clinically indicated. We collected and statistically analyzed patient demographics, initial knee conditions, and surgical details.

**Results:**

Among the patients, 52% required deep MCL release. Notably, patients without soft tissue release exhibited lower initial hip-knee-ankle (HKA) angles, reduced varus-valgus stress test angles, and a greater range of flexion. We identified a predictive threshold HKA angle of 6.250 degrees, demonstrating high sensitivity and specificity for determining the need for deep MCL release.

**Conclusion:**

This study underscores the significance of the initial HKA angle and varus-valgus stress tests in predicting deep MCL release during TKA. The established HKA angle threshold simplifies surgical decision-making, reducing the likelihood of unnecessary soft tissue release.

## Introduction

Total knee arthroplasty (TKA) stands as a cornerstone in the management of severe knee arthritis, demanding precision in alignment, implant placement, and soft tissue balance to secure successful outcomes [1]. Achieving optimal alignment and stability holds paramount importance, not only for clinical functionality but also for the long-term success of TKA. In cases of medial compartment osteoarthritis, the requirement for medial soft tissue release becomes imperative under certain circumstances due to its substantial influence on joint stability and postoperative results [2, 3]. Nevertheless, achieving the right equilibrium in medial soft tissue release poses a delicate challenge; excessive release can lead to joint instability, while insufficient release may result in postoperative discomfort and stiffness [4]. 

In studies involving conventional TKA for varus knees, some have reported that deep medial collateral ligament release is required in all cases [2, 5]. Nevertheless, a study focused on robotic-assisted cementless TKA revealed that only 29.7% of cases needed soft tissue release to achieve balance during mechanical alignment and flexion gap balancing [6]. The existing body of literature on MCL release in TKA has provided valuable insights into the factors influencing soft tissue balancing [7, 8]. A study indicated that pre-operative radiographic knee mechanical axis angle, mechanical varus stress angle, mechanical valgus stress angle, and the sum of the mechanical varus and valgus stress angles were the most influential predictors of reduction osteotomy for soft tissue balancing during TKA [9]. Other studies have found pre-operative distractive stress radiographs to be useful in predicting the extent of medial release [10, 11]. 

Adjusted mechanical alignment (aMA) TKA is a modified surgical technique aiming to achieve a well-balanced knee with minimal soft tissue releases [12]. Unlike traditional mechanical alignment, adjusted mechanical alignment preserves a slight degree of the patient’s native knee deformity by adjusting component position within 3 degrees of varus or valgus from the mechanical axis [13]. Research suggests that adjusted mechanical alignment may potentially reduce the need for extensive soft tissue releases [14]. However, there remains a significant gap in our understanding of the factors influencing MCL release during adjusted mechanical alignment robot-assisted TKA. This study aims to systematically investigate the relationship between preoperative knee data and the need for MCL release in this context. By identifying these factors, we seek to provide evidence-based guidance for orthopedic surgeons to optimize TKA outcomes.

## Materials and methods

This investigation constitutes a retrospective study conducted at a tertiary hospital from January 2023 to July 2023. Patient data were retrieved from the hospital’s electronic database, with approval from the local ethics committee and Institutional Review Board (review number 701/2566), waiving the need for patient consent. The study enrolled patients who underwent primary TKA with robotic assistance for medial compartment osteoarthritis. Cases with incomplete data were excluded, as these resulted from premature termination of the robotic-assisted procedure due to technical issues, such as pin or tracker displacement.

All cases followed a standardized procedural sequence assisted by the robotic surgical assistant ROSA (Zimmer Biomet, Warsaw, IN, USA). Cemented posterior-stabilized TKA prostheses (Zimmer Biomet, Warsaw, IN, USA) were uniformly used. A standard medial parapatellar approach, along with a tourniquet, was consistently employed. Initial surgical steps involved exposing the anterior aspect of the tibia while preserving the deep medial collateral ligament (MCL), followed by excision of proximal and femoral osteophytes. Subsequent steps involved bone registration, assessment of alignment, and soft tissue laxity.

Flexion range of motion was assessed by performing full knee flexion while the hip was positioned at 90 degrees, and varus and valgus stresses were manually applied at 0 and 90 degrees of knee flexion.

Intra-operative planning and adjustments involved setting an alignment goal of achieving mechanical alignment with a Hip-Knee-Ankle (HKA) angle of 0 degrees. This resulted in femoral and tibial bone cuts that were perpendicular to the mechanical axis. However, in cases where the initial evaluation identified an imbalanced gap, adjustments were made to the alignment of the femoral and tibial cuts to achieve an HKA angle within ± 3°, thereby ensuring gap balance. Femoral rotation was adjusted to attain gap balance within a range of 2–5 degrees of external rotation relative to the posterior condylar axis (PCA).

The initial bone cut depth was carefully determined to account for both bone removal and soft tissue tension. Our target was 19 mm in the lateral compartment, allowing for flexibility in the medial compartment. Component selection was guided by the extension gap space, and we proceeded with distal femoral cuts, femoral 4-in-1 bone cuts, and proximal bone cuts.

Following these steps, femoral and tibial trial components were inserted. Alignment, range of motion, and gap balance were then assessed. A deep medial collateral ligament (MCL) release was indicated when the medial gap exceeded the lateral gap by more than 2 mm. Conversely, a superficial MCL release was performed if the medial gap remained narrower than the lateral gap. Final alignment, range of motion, and gap balance were confirmed before implanting the final components. The standard femoral component width is the default size of the implant. A narrower width implant is selected within the same anteroposterior size when there is medial and lateral femoral overhang.

Statistical analysis was conducted using the R-program version 3.2.1 software (R Foundation for Statistical Computing, Vienna, Austria). Categorical variables were analyzed using the Chi-square test, while continuous variables were examined using either the independent samples t-test or the Mann-Whitney U test. The optimal threshold for predicting the necessity of MCL release was determined from the Receiver Operating Characteristic (ROC) curve analysis, with Youden’s Index used as the criterion [15]. A significance level of *p* < 0.05 was employed to establish statistical significance.

The sample size was calculated based on data from Hernandez-Vaquero et al. regarding the femoral-tibial angle and the necessity for soft tissue release in TKA. Hernandez-Vaquero et al. reported proportions of 0.071 and 0.521 for cases achieving a neutral femoral-tibial axis after the varus-valgus stress angle test [16]. Based on a type I error probability of 0.05, a type II error probability of 20%, and a 1:1 ratio, a minimum of 15 patients per group was required. Ultimately, data were collected from approximately 60 patients undergoing surgery at our institution to further minimize selection bias.

## Results

The study enrolled a total of 61 eligible patients, with 52% (32 out of 61 patients) requiring deep release. Within this subgroup, all necessitated deep MCL release, and 2 patients required superficial MCL release. Patients were categorized into two groups: one group without soft tissue release (29 patients) and another group with soft tissue release (32 patients). Demographic data showed no significant differences between the groups. (Table 1)

**Table 1:** Demographic data

Characteristic	Group 1 (Non release) *N* = 29	Group 2 (Release) *N* = 32	*p*-value
Gender (men women) Age (years) Side (left: right) Robotic usage time (minutes)^a^ Polyethylene size Implant (standard : narrow)	2:27 65 (63-73.5) 8:21 33 (29-41.5) 10.241 ± 0.44 14:15	5:27 69 (63.5–77) 19:13 39 (31.5–49) 10.219 ± 0.49 17:15	0.285 0.183 0.013 0.142 0.85 0.705

Represents Mean ± SD, Median (IQR)

^a^ Time from start of bone registration to final implant placement

The analysis of initial knee status revealed that patients without soft tissue release had significantly lower HKA angles, lower varus alignment in both varus and valgus stress tests in the extension position, and lower varus alignment in the varus stress test in the flexion position compared to patients requiring soft tissue release. Additionally, the non-soft tissue release group exhibited a higher range of motion during flexion. (Table 2)

**Table 2:** Initial knee stat

Characteristic	Group 1 (Non release) *N* = 29	Group 2 (Release) *N* = 32	*p*-value
Initial Hip-Knee-Ankle (HKA) angle Minimum Flexion [DEG] Maximum Flexion [DEG] Extension: Medial laxity [mm] Extension: Lateral laxity [mm] Extension: Maximum varus stress [DEG] Extension: Maximum valgus stress [DEG] Flexion: Medial laxity [mm] Flexion: Lateral laxity [mm] Flexion: Maximum varus stress [DEG] Flexion: Maximum valgus stress [DEG]	5 (1.72–6.25) 3.75 ± 4.74 124.4 (119.3-127.5) 2.2 (1.05–3.95) 2.26 ± 1.44 varus 8.5 (5.45–9.75) varus 2.12 ± 2.9 2.22 ± 1.36 3.08 ± 1.92 10.42 ± 3.54 2.03 ± 2.78	9.250 (6.5–11.5) 2.93 ± 6.29 122.25 (116.45–124.6) 2.3 (1.9–3.2) 2.38 ± 1.87 varus 13.2 (9.6-15.35) varus 5.48 ± 2.74 2.65 ± 1.89 3.19 ± 1.4 14.52 ± 3.99 3.36 ± 2.56	< 0.001 0.575 0.016 0.613 0.777 < 0.001 < 0.001 0.317 0.796 < 0.001 0.56

Represents Mean ± SD, Median (IQR)

Patients in the soft tissue release group exhibited a slightly higher varus position of the tibial component and a slightly higher varus alignment in the HKA angle, though these differences were not statistically significant. No significant differences were observed between the groups regarding the position of the femoral component and soft tissue laxity during stress tests in both flexion and extension positions. (Table 3)

**Table 3:** Postoperative stat

Characteristic	Group 1 (Non release) *N* = 29	Group 2 (Release) *N* = 32	*p*-value
Femoral component -Flexion -Varus/Valgus alignment [DEG] -Rotation from PCA [DEG] Tibial component -Slope Validation [DEG] -Varus/Valgus alignment [DEG] Post implant HKA angle [DEG] Extension: Medial laxity [mm] Extension: Lateral laxity [mm] Flexion: Medial laxity [mm] Flexion: Lateral laxity [mm]	2.43 ± 0.7 varus 0.5 (0–1) 4 (3-4.5) 3.79 ± 1.28 varus 1.38 ± 1.12 1.98 ± 1.22 1.3 (1-1.5) 1.4 (1.1–1.6) 0.94 ± 0.31 1 (0.7–1.25)	2.54 ± 0.68 varus 0.5 (0–1) 4 (3-4.5) 3.73 ± 0.81 Varus 1.96 ± 1.09 2.5 ± 1.07 1.4 (1-1.9) 1.4 (1.1–1.85) 1.01 ± 0.43 1 (0.7–1.15)	0.527 0.758 0.806 0.831 0.44 0.81 0.737 0.838 0.485 0.988

Represents Mean ± SD, Median (IQR)

**Fig. 1:**
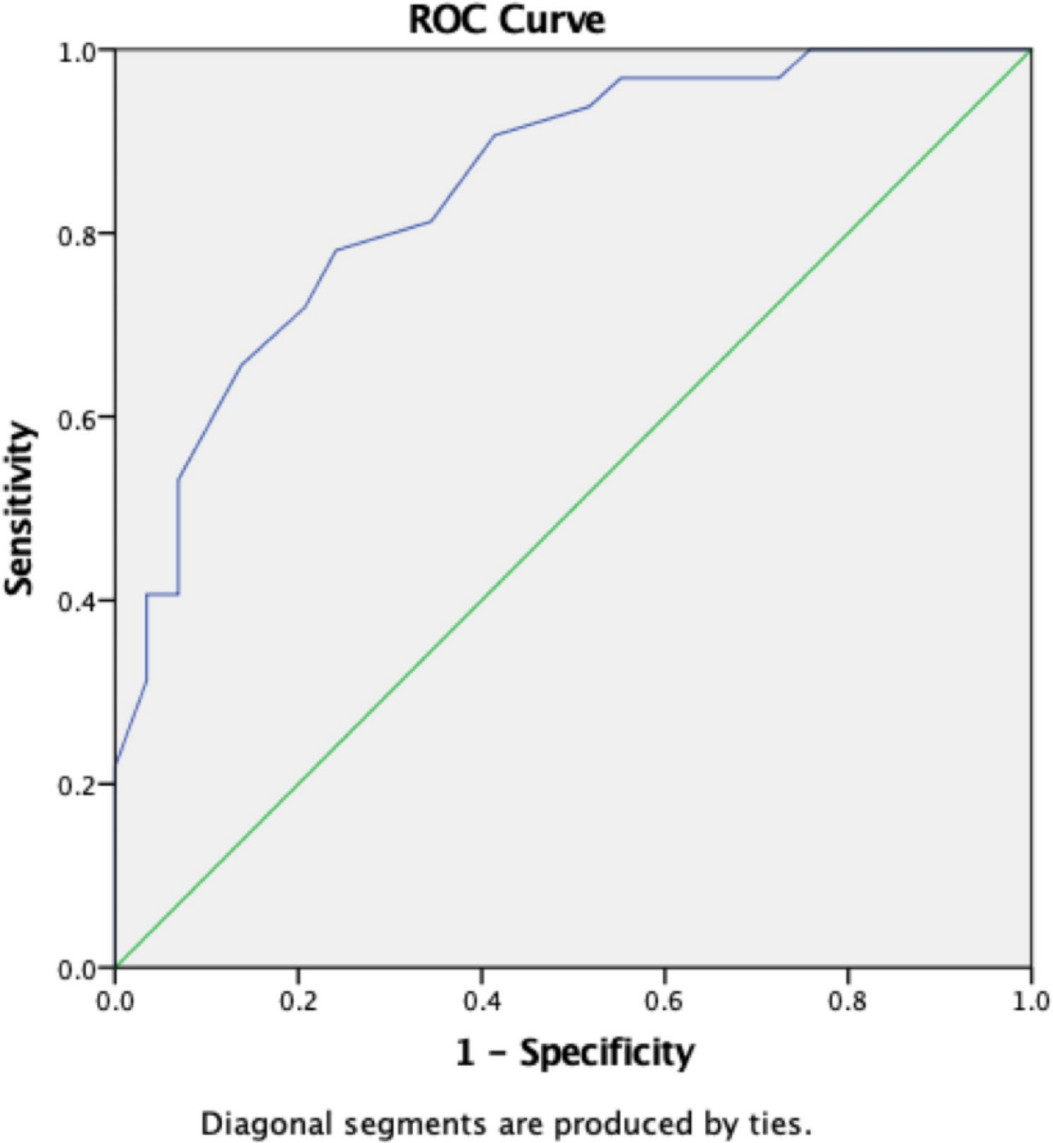
Receiver Operating Characteristic (ROC) curve analysis for determining the optimal Hip-Knee-Ankle (HKA) angle threshold to predict the necessity of deep medial collateral ligament (MCL) release

The receiver operating characteristic (ROC) curve analysis was conducted to determine the optimal threshold of the HKA angle for predicting the necessity of deep MCL release in robotic-assisted TKA. (Fig. 1) In our study, the highest Youden’s Index value was observed at an HKA angle threshold of 6.25 degrees, with a corresponding sensitivity of 0.781 and specificity of 0.759. We found that an HKA angle greater than 6.25 degrees was predictive of the necessity for MCL release to achieve proper gap balancing, as it indicated a medial gap exceeding the lateral gap by more than 2 mm.

## Discussion

Properly balancing soft tissue in total knee arthroplasty (TKA) is crucial for the procedure’s success and longevity [16]. While existing literature has illuminated factors affecting soft tissue release in conventional TKA, there remains a gap in our understanding when it comes to data obtainable from adjusted mechanical alignment robotic-assisted TKA systems. Our study aimed to bridge this gap by exploring the relationship between various factors and the necessity for medial soft tissue release in patients with medial compartment osteoarthritis.

Our findings highlighted several parameters associated with the requirement for medial soft tissue release, including the initial HKA angle, maximum flexion degree, maximum varus and valgus stress test angles in the extension position. Significantly, our research also identified a potential threshold value for the HKA angle that can serve as a predictive indicator for the necessity of medial soft tissue release.

One noteworthy observation from our study was that patients who did not require soft tissue release exhibited lower HKA angles, indicating a more favorable preoperative knee alignment. This aligns with the findings of other studies, where a larger preoperative knee mechanical axis angle was indicative of a greater extent of medial soft tissue release [2, 9]. Ahn JH et al. reported that the sum of mechanical varus and valgus stress angles is a significant risk factor for reduction osteotomy, [9] while Sim JA et al. noted that the extent of medial release increases with the degree of varus deformity observed in preoperative distractive stress radiographs. [10] Another study conducted by Hernandez-Vaquero D et al. reported that if a patient is able to achieve a femoral-tibial angle of 0 degrees during the varus-valgus stress test, there is a lower incidence of requiring medial soft tissue release [16]. 

Our study identified a threshold of 6.25 degrees in the HKA angle, which demonstrated strong predictive accuracy for the necessity of medial soft tissue release, with sensitivity and specificity values of 0.781 and 0.759, respectively. However, when compared to the study by Toyooka S et al., it becomes evident that males with a preoperative varus deformity greater than 5.3 degrees and females with a deformity exceeding 9.1 degrees had a higher incidence of requiring extensive medial release to achieve neutral alignment [17]. 

Several limitations must be acknowledged in our study. Firstly, the retrospective design introduces the potential for selection bias and uncontrolled confounders. Although appropriate statistical methods were used to mitigate these issues, prospective studies with standardized criteria could yield more rigorous evidence. A second limitation of our study is the potential variability in the manual application of force during stress tests and motion assessments. While the computer navigation system provided accurate measurements, the force applied during flexion, varus, and valgus stress tests may not have been consistently accurate. To minimize this variability, all stress tests and assessments were performed by a single surgeon, ensuring consistency in technique. Lastly, our study’s focus on preoperative and intraoperative data lacks long-term follow-up to assess the sustained clinical implications of deep MCL release decisions. Addressing this limitation would involve extended follow-up to evaluate the durability and impact of these decisions on patient outcomes.

## Conclusion

In conclusion, our study revealed a significant association between the initial HKA angle, varus-valgus stress test angle, and the necessity for medial soft tissue release during TKA. A threshold HKA angle of 6.250 degrees, demonstrating high sensitivity and specificity, emerged as a reliable predictor for avoiding medial soft tissue release. These findings emphasize that not all patients with medial compartment osteoarthritis require soft tissue release. By identifying patients with favorable knee alignment, surgeons can potentially reduce surgical invasiveness, expedite recovery, and enhance patient outcomes. While this study provides valuable insights, further research is warranted to validate these findings in a larger and more diverse population.

## Data Availability

No datasets were generated or analysed during the current study.
